# Conjunctival microcirculatory blood flow is altered but not abolished in brain dead patients: a prospective observational study

**DOI:** 10.1186/s12883-016-0618-z

**Published:** 2016-07-11

**Authors:** Tomas Tamosuitis, Andrius Pranskunas, Neringa Balciuniene, Vidas Pilvinis, E. Christiaan Boerma

**Affiliations:** Department of Intensive Care Medicine, Lithuanian University of Health Sciences, Eiveniu str. 2, LT-50009 Kaunas, Lithuania; Department of Intensive Care Medicine, Medical Center Leeuwarden, Henri Dunantweg 2, Leeuwarden, 8901 BR The Netherlands; Department of Translational Physiology, Academic Medical Center, Amsterdam, The Netherlands

**Keywords:** Brain death, Conjunctiva, Microcirculation

## Abstract

**Background:**

The conjunctival microcirculation has potential as a window to cerebral perfusion due to related blood supply, close anatomical proximity and easy accessibility for microcirculatory imaging technique, such as sidestream dark field (SDF) imaging. Our study aims to evaluate conjunctival and sublingual microcirculation in brain dead patients and to compare it with healthy volunteers in two diametrically opposed conditions: full stop versus normal arterial blood supply to the brain.

**Methods:**

In a prospective observational study we analyzed conjunctival and sublingual microcirculation using SDF imaging in brain dead patients after reaching systemic hemodynamic targets to optimize perfusion of donor organs, and in healthy volunteers. All brain death diagnoses were confirmed by cerebral angiography. Microcirculatory images were obtained and analyzed using standardized published recommendations. Study registered at ClinicalTrials.gov, number NCT02483273.

**Results:**

Eleven brain dead patients and eleven apparently healthy controls were enrolled in the study. Microvascular flow index (MFI) of small vessels was significantly lower in brain dead patients in comparison to healthy controls in ocular conjunctiva (2.7 [2.4–2.9] vs. 3.0 [2.9–3.0], *p* = 0.01) and in sublingual mucosa (2.8 [2.6–2.9] vs. 3.0 [2.9–3.0], *p* = 0.02). Total vessel density (TVD) and perfused vessel density (PVD) of small vessels were significantly lower in brain dead patients in comparison to healthy controls in ocular conjunctiva (10.2 [6.6–14.8] vs. 18.0 [18.0–25.4] mm/mm^2^, *p* = 0.001 and 5.0 [3.5–7.3] vs. 10.9 [10.9–13.5] 1/mm, *p* = 0.001), but not in sublingual mucosa.

**Conclusion:**

In comparison to healthy controls brain dead patients had a significant reduction in conjunctival microvascular blood flow and density. However, the presence of conjunctival flow in case general cerebral flow is completely absent makes it impossible to use the conjunctival microcirculation as a substitute for brain flow, and further research should focus on the link between the ocular microcirculation, intracranial pressure and alternative ocular circulation.

## Background

Over the last decade improved microcirculatory imaging techniques such as sidestream dark field (SDF) videomicroscopy have allowed the direct observation of the microcirculation to assess capillary flow and density in humans in various clinical conditions [1–4]. Clinical studies have shown that microvascular alterations of sublingual microcirculation are associated with inverse outcomes [2, 5] and may direct therapeutic approaches [6–8]. For practical reasons the microcirculation is observed in the sublingual area in the vast majority of publications. SDF imaging of ocular conjunctiva, however, has also been tested successfully [9] and has the potential to be used as a surrogate measurement of cerebral perfusion. The human brain is a highly perfused organ with blood supply mainly dependent on the carotid circulation, branching off into two pairs of arteries finally interconnecting into the Circle of Willis [10]. The human ocular circulation is mainly supplied by ophthalmic artery (OA) which is a first major branch of internal carotid artery (ICA) with little contribution from the external carotid artery [11, 12]. Close anatomical proximity to the brain, common root of blood supply and availability for direct evaluation makes ocular conjunctiva a tempting window for a quick, noninvasive and dynamic assessment of cerebral perfusion. Schaser and colleagues [13] analyzed conjunctival microcirculation using orthogonal polarization spectral (OPS) microscopy during carotid surgery aiming to recognize early cerebral ischemia caused by ICA clamping. The authors came to the conclusion that conjunctival OPS imaging enables continuous monitoring of shunt efficiency during carotid endarterectomy and the direct observation of compensatory vascular changes in the terminal region of the ICA. In a study published in abstract an inverse relationship between SDF-derived conjunctival microvascular flow and rise in intracranial pressure (ICP) was observed in head trauma patients [14]. In addition, Doppler-based measurements of OA and central retinal artery have been used to assess intracranial perfusion pressure [15, 16].

However, despite the obvious link between the cerebral and ocular perfusion scientific data to link ocular microcirculatory perfusion with critical states of brain hypoperfusion remain scarce. Our study aims to evaluate conjunctival and sublingual microcirculation in brain dead patients and to compare it with healthy volunteers in two diametrically opposed conditions: full stop versus normal arterial blood supply to the brain.

## Methods

### Setting

This prospective single-center observational study was performed during a 9 months period in 2013/2014 in an 18 bed neurosurgical ICU in a tertiary teaching hospital.

### Protocol and data collection

All study patients were included in the trial within first 24 h after final diagnosis of brain death was certified using clinical symptoms and ancillary test (cerebral angiography) according to Lithuanian Health ministry regulations and international guidelines [17, 18]. Cerebral angiography (GE INNOVA, USA) was carried out by two experienced interventional radiologists performing more than 1000 interventions annually. Sepsis, anemia and hypoxia were excluded by clinical and laboratory evaluation prior to the initiation of the study protocol.

All brain dead patients were equipped with a central venous catheter and arterial femoral PiCCO® catheter (PULSION Medical systems, Munich, Germany) as standard monitoring for multi-organ donors. Patients were managed according to the local institutional multi-organ donor protocol based on international recommendations [18–20]. The study protocol was initiated after systemic hemodynamic, core temperature and electrolyte targets, aiming for optimal homeostasis of internal organs, had been achieved. The following data were recorded at baseline: general characteristics, systemic hemodynamic parameters, routine laboratory tests, arterial blood gases, sublingual and bilateral conjunctival SDF imaging.

The control group consisted of healthy volunteers with no reported ocular pathology. After a 30 min resting time hemodynamic parameters and ocular and sublingual SDF images were taken. None of the control group individuals was locally anaesthetized or did report any discomfort related to the ocular SDF-imaging.

### Videomicroscopic measurements and analysis

Sublingual and conjunctival microcirculation images were obtained using SDF videomicroscopy (Microscan®, Microvision Medicals, Amsterdam, the Netherlands). We followed published expert recommendations for quality and analysis of obtained images [21]. Images of microcirculation were taken from at least three different points in each location and recorded for at least 10–20 s, avoiding pressure artifacts and after gentle removal of saliva in the sublingual area or tears in the conjunctival area. Data were recorded at the hard disk-drive of a personal computer using AVA 3.0v software (Microvision Medical, Amsterdam, Netherlands) for further analysis [22].

Video clips were blindly analyzed off-line by two investigators in random order to prevent coupling. Each image was divided into four equal quadrants. Quantification of flow (no flow: 0; intermittent flow: 1; sluggish flow: 2; continuous flow: 3) was scored per quadrant, for each vessel diameter cohort (small: 10–20 μm; medium: 21–50 μm; large: 51–100 μm). The Microvascular Flow Index (MFI) was calculated as the sum of each quadrant score divided by the number of quadrants in which the vessel type was visible. The final MFI was averaged over a maximum of 12 quadrants (three regions, four quadrants per region) derived from the overall flow impressions of all vessels with a particular range of diameter in a given quadrant. Calculation of total (small) vessel density (TVD) was performed with the AVA 3.0® software package (Microvision Medical, Amsterdam, The Netherlands) [22] using a cutoff diameter for small vessels of <20 μm. After stabilization of the images, perfused (small) vessel density (PVD) was calculated as the number of crossings with (perfused) (small) vessels per total length of three equidistant horizontal and three equidistant vertical lines.

### Statistical analysis

Data were analyzed with Statistical Package for Social Sciences (SPSS 22 for Windows, Chicago USA). With respect to small numbers, data are presented as the median [25th–75th percentiles] and analyzed with non-parametric tests. A *p* value of < 0.05 was considered significant.

## Results

### Demographic and systemic hemodynamic data

Eleven brain dead patients and eleven healthy control individuals were recruited in this study. Both groups did not differ in main demographic and hemodynamic parameters (Table 1).

**Table 1:** Baseline demographic and clinical characteristics (*n* = 11)

	Brain dead	Control	*p* value
Gender, male/female, n	9/2	6/5	0.18
Age (years)	45 [32–63]	36 [26–43]	0.15
Heart rate (beats/min)	84 [73–97]	78 [68–90]	0.34
Mean arterial pressure (mmHg)	89 [78–93]	74 [68–92]	0.08

Data are presented as median [interquartile range] unless stated otherwise

The brain dead group consisted of five patients with traumatic brain injury, three with intracerebral hemorrhage and three with subarachnoid hemorrhages due to rupture of a cerebral aneurysm. Nine brain dead patients required vasopressor support: six managed with norepinephrine, two with dopamine and one required both. Median values of vasopressor support were low to intermediate and donor protocol driven cardiac output was reached in all cases (Table 2).

**Table 2:** Clinical data of brain dead patients (*n* = 11)

Main disease, n (%)	
Traumatic brain injury Intracerebral hemorrhage Subarachnoid hemorrhage	5 (46) 3 (27) 3 (27)
Vasopressors, (n) Dopamine, n; (μg/kg/min) Norepinephrine, n; (μg/kg/min)	9 3; 8 [2.0-9.0] 7; 0.08 [0.04-0.1]
Cardiac index (l/min/m^2^)	3.2 [2.8-4.0]
Hb concentration (g/l)	129 [105–141]
WBC count (×10^9^ / l)	10.8 [9.4-12.6]
CRP (g/l)	15 [12–76]
pH	7.36 [7.32-7.40]
pO_2_ (mmHg)	145 [75–200]

Data are presented as median [interquartile range] unless stated otherwise, *Hb* hemoglobin, *WBC* white blood cell, *CRP* C reactive protein, *pO*_*2*_ = oxygen partial pressure

### Microcirculation data

**Fig. 1:**
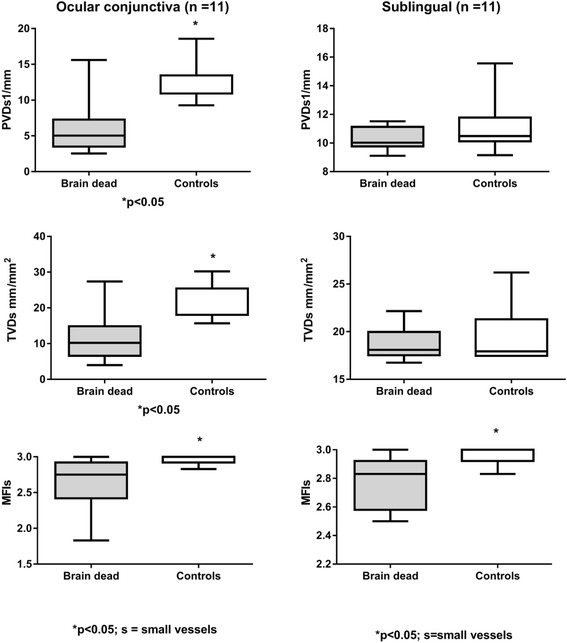
Conjunctival and sublingual microcirculation data of brain dead patients and control group TVD- total vessel density; PVD- perfused vessel density; MFI- microvascular flow index

The conjunctival and sublingual microcirculation data are presented in Fig. 1. The MFI of small vessels was significantly lower in brain-dead patients in comparison with healthy controls in ocular conjunctiva (2.75 [2.40 – 2.90] vs. 3.00 (3.00 – 3.00), *p* = 0.01) and in sublingual mucosa (2.83 [2.60 – 2.90] vs. 3.00 [3.00 – 3.00], *p* = 0.02). TVD and PVD of small vessels were significantly lower in brain-dead patients in comparison with healthy controls in the ocular conjunctiva (10.2 [6.6 – 14.8] vs. 18.0 [18.0 – 25.4] mm/mm^2^, *p* = 0.001 and 5.0 [3.5 – 7.3] vs. 10.9 [8.5 to 13.5] l/mm, *p* = 0.001), but there was no difference in sublingual mucosa (18.1 [16.7–22.2] vs. 17.9 [17.5–26.2] mm/mm^2^, *p* = 0.76 and 10.0 [9.1–11.5] vs. 10.5 [9.2–15.6] 1/mm, *p* = 0.09, respectively).

**Fig. 2:**
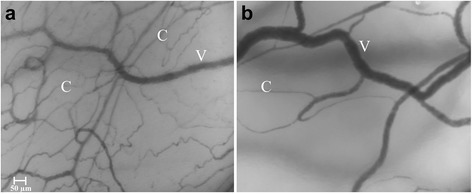
Microscopic images of the conjunctival microcirculation in control group (**a**) and brain dead group (**b**). C - capillaries, V - venules

Digital images of the conjunctival microcirculation in brain dead and healthy individuals are presented in Fig. 2.

## Discussion

To our knowledge this is the first study in which the ocular and sublingual microcirculation is directly observed in brain dead patients. We were able to demonstrate statistically significant differences in perfused vessel density and microvascular flow of small vessels in the conjunctival and sublingual microcirculation in brain dead patients in comparison with healthy control. However, most strikingly, conjunctival microvascular blood flow was clearly present in all brain dead patients, although (by definition) overall cerebral blood flow was absent during angiography.

These observations raise a number of questions. Is conjunctival/ocular blood flow fully dependent on ICA supply? And, if so, to what extent are the observed conjunctival microcirculatory alterations the result of reduced cerebral blood flow, as opposed to disruption of endothelial integrity during brain death? The fact that conjunctival microvascular blood flow is preserved to a large extend during absence of flow in the ICA may be in line with an alternative ocular blood supply. Schaser et al. [13] demonstrated that ICA clamping reduces capillary density and red blood cells velocity in the ipsilateral conjunctiva in carotid artery surgery patients. However, these authors also noticed that the conjunctival microcirculation by no means was halted to a standstill, which they explained by the development of a collateral compensatory circulation. However, in brain dead humans the acute timeline is unlikely to allow for neovascularisation. Alternatively, conjunctival vascularisation is not fully ICA dependent. In retrospect we found evidence of preserved circulation via the OA in two out of three brain dead patients with lateral angiographic imaging. We were unable to evaluate ophthalmic circulation in other patients, since the routine position for cerebral pan-angiography is anterior-posterior. This position is not ideal for the detection of contrast filling of the OA. It is conceivable that parts of the external carotid artery circulation contribute to the conjunctival microcirculation. We expect to perform lateral imaging during cerebral angiography in the future to clarify different ocular blood supply routes in brain dead patients.

Overall cerebral blood flow is determined by mean arterial pressure and intracranial pressure [23]. Knowing that the ophthalmic artery lies in the subdural space while traveling intracranialy suggests that changes in ICP should be reflected in flow in the OA and possibly influence the circulation in terminal branches in the bulbar conjunctiva. Indeed, data in abstract form showed that in traumatic brain injury patients conjunctival capillary flow depended on the grade of increased ICP and subsequent decrease in cranial perfusion pressure [14, 24]. Interesting, recently a study has been published regarding non-invasive ICP monitoring using OA flow measurements with two-depth transcranial Doppler, assuming that intracranial segment of OA is compressed by raised ICP and extracranial part of OA by pressure applied on the orbit [15]. Results of OA transcranial Doppler measurements proved to be accurate in comparison with ICP measurements via lumbar puncture. However, ICP values did not reach a critical point. Nevertheless, this paper initiated vivacious responses from other investigators with contra-arguments whether external segment of the OA is totally influenced by orbital pressure only [25]. In addition, Miller et al. [16] demonstrated the effect of elevated ICP on the reduction in central retinal artery flow velocities, measured by spectral Doppler imaging. These results support the hypothesis of a close interconnection between ICP driven cerebral perfusion and the ocular circulation, analyzed at various anatomical levels of the intracranial part of OA.

The fact that alterations in sublingual microvascular density were not significantly different from healthy controls, in contrast to the conjunctival microcirculation, suggests the influence of ICP. However, blood supply of the sublingual area is mainly dependent on the external carotid artery and should not be influenced by ICP. Nevertheless, sublingual microvascular blood flow is equally altered in brain dead patients, suggesting additional factors. Brain death is typically followed by hemodynamic instability and endothelial dysfunction, with or without a prior autonomic storm [26, 27].

The vast majority of our patients needed vasopressor therapy. It is known that in human sepsis microcirculatory alterations occur independently of central hemodynamic changes and that there are differences in microcirculatory response between the sublingual and conjunctival area [9, 28]. In addition sublingual microvascular alterations may also reflect intracerebral pathology. Khalilzada and colleagues [29] stated that sublingual reduction of functional capillary density is statistically more frequent in patients with small vessel disease than in patients with large vessel disease or healthy volunteers when studying stroke patients. Another sublingual microcirculation study in stroke patients detected compromised glycocalyx barrier properties, consistent with impaired endothelial function [30]. The contralateral impairment of conjunctival microvascular blood flow after ICA clamping [13] may also be in line with the idea that intracerebral pathology may be reflected in microvascular blood flow of other parts of the body.

Our study has some limitations. We did not perform angiography of the ophthalmic artery circulation. Nor did we perform cerebral angiography in our control group for obvious reasons. Literature suggests variations in common arterial blood supply via ophthalmic artery in healthy individuals [11].

## Conclusion

We were able to demonstrate statistically significant differences in perfused vessel density and microvascular flow of small vessels in the conjunctival and sublingual microcirculation in brain dead patients in comparison with healthy control. However, the presence of conjunctival microvascular flow in case of absent general cerebral flow makes it impossible to use direct in-vivo microscopy of the conjunctival microcirculation as a substitute for brain flow. Further research should focus on the link between ocular microcirculatory alteration and cerebral pathology, including the link between intracranial pressure and (alternative) ocular circulation.

## Abbreviations

CO, cardiac output; ICP, intracranial pressure; MAP, mean arterial pressure; MFI, microvascular flow index; OA, ophthalmic artery; OPS, orthogonal polarization spectral; PPV, proportion of perfused vessels; PVD, perfused vessel density total vessel density (TVD); SDF, sidestream dark field; TBI, traumatic brain injury; TCD, transcranial doppler
